# Therapeutic effect of anti-C-X-C motif chemokine 10 (CXCL10) antibody on C protein-induced myositis mouse

**DOI:** 10.1186/ar4583

**Published:** 2014-06-17

**Authors:** Jinhyun Kim, Ji Yong Choi, Sung-Hye Park, Seung Hee Yang, Ji Ah Park, Kichul Shin, Eun Young Lee, Hiroshi Kawachi, Hitoshi Kohsaka, Yeong Wook Song

**Affiliations:** 1 Department of Internal Medicine, Seoul National University College of Medicine, 28 Yongon-dong, Chongno-gu, Seoul 110-744, Korea; 2 Department of Pathology, Seoul National University College of Medicine, Seoul, Korea; 3 Seoul National University Kidney Research Institute, Seoul, Korea; 4 Department of Cell Biology, Institute of Nephrology, Niigata University Graduate School of Medical and Dental Sciences, Niigata, Japan; 5 Department of Medicine and Rheumatology, Graduate School of Medical and Dental Sciences, Tokyo Medical and Dental University, Tokyo, Japan; 6 Department of Molecular Medicine and Biopharmaceutical Sciences, Graduate School of Convergence Science and Technology and College of Medicine, Medical Research Center, Seoul National University, Seoul, Korea

## Abstract

**Introduction:**

C-X-C motif chemokine 10 (CXCL10) is a chemokine that plays a critical role in the infiltration of T cells in autoimmune diseases and is reported to be expressed in muscle tissue of polymyositis. To determine the therapeutic efficacy of CXCL10 blockade, we investigated the role of CXCL10 and the effect of anti-CXCL10 antibody treatment in C protein-induced myositis (CIM), an animal model of polymyositis.

**Methods:**

CIM was induced with human skeletal muscle C protein fragment in female C57BL/6 mice. Immunohistochemistry of CXCL10 and C-X-C motif chemokine receptor 3 (CXCR3) and measurement of serum CXCL10 were performed. Cell surface markers and interferon-gamma (IFN-γ) and tumor necrosis factor-alpha (TNF-α) in CIM lymph node cells was investigated by flow cytometry. Mice with CIM were treated with anti-CXCL10 antibody or control antibody (anti-RVG1) and the inflammation in muscle tissue was assessed.

**Results:**

Immunohistochemistry showed increased expression of CXCL10 and CXCR3 in the inflammatory lesions of muscle in CIM. Especially, CD8+ T cells invading myofiber expressed CXCR3. Serum level of CXCL10 was increased in CIM compared to the level in normal mice (normal mouse, 14.3 ± 5.3 pg/ml vs. CIM, 368.5 ± 135.6 pg/ml, *P* < 0.001). CXCR3 positivity in CD8+ T cells was increased compared to that of CD4+ T cells in the lymph node cells of CIM (CXCR3+ among CD8+ T cell, 65.9 ± 2.1% vs. CXCR3+ among CD4+ T cell, 23.5 ± 4.7%, *P* <0.001). Moreover, IFN-γ+ cells were increased among CXCR3+CD8+ T cells compared to CXCR3–CD8+ T cells (CXCR3+CD8+ T cell, 28.0 ± 4.2% vs. CXCR3-CD8+ T cell, 9.5 ± 1.5%, *P* = 0.016). Migration of lymph node cells was increased in response to CXCL10 (chemotactic index was 1.91 ± 0.45). CIM mice treated with anti-CXCL10 antibody showed a lower inflammation score in muscles than those with anti-RVG1 (median, anti-CXCL10 treatment group, 0.625 vs. anti-RVG1 treatment group, 1.25, *P* = 0.007).

**Conclusions:**

CXCL10/CXCR3 expression was increased in the inflammation of CIM model and its blockade suppressed inflammation in muscle.

## Introduction

Chemokines are 8- to 10-kDa proteins with 20 to 70% amino acid sequence homology and produce chemotactic activity in various cells, especially immune cells [1]. To date, approximately 50 different chemokines and at least 20 different receptors have been identified [2]. Among them, C-X-C motif chemokine 10 (CXCL10; also known as interferon-γ inducible protein-10, IP-10) is a chemokine that potentially plays a role in the immunopathogenesis of autoimmune disease such as rheumatoid arthritis, systemic lupus erythematosus, systemic sclerosis, and idiopathic inflammatory myopathy (IIM) [3-6]. It can be secreted by various cell types, such as, monocytes, neutrophils, endothelial cells, keratinocytes, fibroblasts, mesenchymal cells, dendritic cells, thyrocytes, cardiac cells, and astrocytes in diverse conditions [7-9]. CXCL10 binds to its receptor CXC chemokine receptor 3 (CXCR3), and regulates immune responses by activation and recruitment of immune cells. CXCR3 is a seven-transmembrane, G protein-coupled cell surface chemotactic receptor for C-X-C motif chemokine 9 (CXCL9; also known as monokine induced by interferon-γ, MIG), CXCL10/IP-10, and C-X-C motif chemokine 11 (CXCL11; also known as interferon-inducible T cell α chemoattractant, I-TAC), and has been suggested to play an important role in lymphocyte trafficking, with preferentially activated T cells [10]. Originally it was cloned from T cells [11], but now it is clear that CXCR3 is expressed on activated T cells, natural killer cells, monocytes, dendritic cells, endothelial cells, and microglia [12-14]. These CXCR3+ cells can produce IFN-γ, which can induce CXCL10 in turn. This crosstalk between immune cells and resident cells may potentially activate the immune system [15]. Previous reports suggested that chemokines not only play an important role in lymphocyte recruitment to inflammatory sites but also participate in T cell activation [16]. Especially, CXCL10 is implicated in autoimmune pathogenesis through the initiation and maintenance of T helper 1 (Th1) response [17].

IIMs are rare autoimmune diseases characterized by proximal muscle weakness, elevated muscle enzymes, abnormal electromyographic findings, and inflammation or vasculopathy in muscle tissue. IIMs are composed of many kinds of diseases with idiopathic muscle inflammation and include dermatomyositis, polymyositis, and sporadic inclusion body myositis [18]. In polymyositis and inclusion body myositis, non-necrotic muscle fibers are actively invaded by autoaggressive macrophages and cytotoxic T cells [18,19]. Despite recent advances in immunosuppression, the treatment of IIM is not satisfactory. Although many kinds of treatment including high-dose corticosteroid, intravenous immunoglobulin, azathioprine, cyclophosphamide or other immunosuppressant are available, some patients do not respond to these treatments, especially, when they have lung involvement [19,20].

Chemokines are also known to play an essential role in sustaining the inflammation associated with IIM. In previous reports on IIMs, CXCL10 was abundantly expressed on macrophages and T cells in polymyositis, inclusion body myositis and dermatomyositis whereas CXCL9 and CXCL11 were not altered compared to the control [6,21]. Strong CXCR3 expression has also been observed in the majority of T cells in both polymyositis and dermatomyositis [6,21]. The above data suggest that the CXCL10/CXCR3 interaction in particular may be a potential therapeutic target in IIM.

In an animal model of human polymyositis, C protein-induced myositis (CIM), muscle damage is caused by CD8+ T cell [22], which is similar to the mechanism of damage in human polymyositis [23]. The purpose of this study was to determine the therapeutic efficacy of anti-CXCL10 antibody in the CIM model. First, the expression of CXCL10 and CXCR3 in C-protein induced myositis mice was investigated. Second, the functional aspect of CXCR3-positive cells was studied, and last, the change in muscle inflammation was evaluated after administration of the anti-CXCL10 antibody.

## Methods

### C-protein-induced myositis model

C57BL/6 mice were purchased from OrientBio (Sungnam, Korea). Female mice, ages 8 to 10 weeks, were immunized intradermally with 200 μg of the C-protein fragments emulsified in complete Freund’s adjuvant (CFA) containing 100 μg of heat-killed *Mycobacterium butyricum* (Difco, Franklin Lakes, NJ, USA) [22]. The immunogens were injected at multiple sites of the back and foot pads, and 250 ng of pertussis toxin (PT) (Sigma-Aldrich, St Louis, MO, USA) diluted with 0.03% Triton X was injected intraperitoneally at the same time. CIM mice were treated with anti-CXCL10 antibody or anti-RVG1 (mouse anti-rotavirus IgG1) antibody (n=17 per group). These antibodies were obtained from mouse ascites after intraperitoneal injection of hybridoma cells producing monoclonal anti-CXCL10 or anti-RVG1 antibody as described previously [24]. Another 17 CIM mice were observed without any treatment. Mice were immunized with C-protein at day 0 and treated by injecting monoclonal antibody 200 μg in 100 μL PBS intraperitoneally every other day from day 8 till day 20. Three weeks after induction, mice were sacrificed and sera, spleens and proximal muscles (hamstring and quadriceps) of both hind legs were harvested. Hematoxylin and eosin-stained 10-μm sections of the proximal muscles were examined histologically for the presence of mononuclear cell infiltration and necrosis of muscle fibers. The histologic severity of inflammation in each muscle block was graded as follows: grade 1 = involvement of a single muscle fiber; grade 2 = a lesion involving 2 to 5 muscle fibers; grade 3 = a lesion involving 6 to 15 muscle fibers; grade 4 = a lesion involving 16 to 30 muscle fibers; grade 5 = a lesion involving 31 to 100 muscle fibers; and grade 6 = a lesion involving >100 muscle fibers. When multiple lesions with the same grade were found in a single muscle section, 0.5 of a point was added to the grade. Histologic grading was modified from the article by Sugihara *et al*. [22]. All experiments were done under specific pathogen-free conditions. The experiment was approved by the Institutional Animal Care and Use Committee in Seoul National University Hospital.

### Immunohistochemistry

Immunohistochemical staining for the presence of CXCL9, CXCL10, CXCL11 or CXCR3 was performed according to the manufacturer’s protocol based on the conventional streptavidin-biotin-peroxidase method. Representative sections of 3-μm thickness of paraffin-embedded muscle tissue were rehydrated after deparaffinization by xylene. Antigen retrieval was performed and the sections were washed with citrate buffer. Then, the sections were immersed in 3% H_2_O_2_ for 10 minutes to inhibit endogenous peroxidase activity and washed three times by PBS over the course of 5 minutes. Then, the sections were incubated with various primary antibodies. The primary antibodies were as follows: anti-CXCL9, anti-CXCL10, anti-CXCL11 (Abcam, Cambridge, UK; 1:200) or anti-CXCR3 (Invitrogen, Carlsbad, CA, USA; 1:200). Antigen retrieval was performed by boiling in citrate buffer: 3,3-diaminobenzidine tetrahydrochloride (DAB) was used as a chromogen. Counterstaining with Meyer’s hematoxylin stain followed.

Cryostat-frozen sections (8 μm) were also used for detection of CXCR3. The sections fixed in cold acetone were stained overnight at 4°C with mouse anti-mouse CD4 (Abcam; 1:100), rat anti-mouse CD8a (Santa Cruz Biotechnology, Santa Cruz, CA, USA; 1:100), rat anti-mouse F4/80 (AbD Serotec, Kidlington, UK; 1:100), rabbit anti-mouse CXCR3 (Invitrogen; 1:100) with blocking reagents. A second layer of Alexa Fluor 555-conjugated anti-rabbit, Alexa Fluor 488-conjugated anti-mouse, and Alexa Fluor 647-conjugated anti-rat antibody (all antibodies were purchased from Molecular Probes, Eugene, OR, USA; 1:100) were used as secondary antibodies, respectively. All sections were washed and incubated for an additional 5 minutes with 4′-6-diamidino-2-phenylindole (DAPI, Molecular Probes) for counterstaining. For negative control, primary antibodies were omitted. The bound antibodies were visualized using LSM510 META confocal laser microscopy (Carl Zeiss, Jena, Germany).

### Flow cytometry

Splenocytes of normal mice and CIM mice, and inguinal lymph node cells of CIM mice were harvested. The splenocytes were purified by Ficoll-gradient methods. The cells were enumerated, and 5 × 10^5^ cells were incubated with Fc Block™ (1 μg/mL; BD Bioscience, San Jose, CA, USA). Staining of the cells was performed with the following antibodies: peridinin chlorophyll(PerCP)-labeled anti-mouse CD3 (BD Bioscience), phycoerytherin(PE)-labeled anti-mouse CXCR3, allophycocyanin(APC)-labeled anti-mouse CXCR3, fluorescein isothiocyanate(FITC)-labeled anti-mouse CD4, FITC-labeled anti-mouse CD8, PE-Cy5-labeled anti-mouse F4/80, APC-labeled anti-mouse B220, PE-labeled anti-mouse interferon-gamma (IFN-γ), or PE-labeled anti-mouse TNF-α. All antibodies were purchased from eBioscience (San Diego, CA, USA).

For intracellular cytokine staining, 5 × 10^5^ lymph node cells were plated in Roswell Park Memorial Institute medium (RPMI) 1640 supplemented with 10% fetal calf serum, 100 U penicillin/mL and 100 μg/mL streptomycin (Gibco, Carlsbad, CA, USA). Cultures were incubated with phorbol 12-myristate 13-acetate (PMA; 100 ng/mL; Sigma-Aldrich) plus ionomycin (500 ng/mL; Sigma-Aldrich) in the presence of brefeldin A (10 μg/mL; BD Pharmingen, San Diego, CA, USA) for 4 hours at 37°C. After stimulation, cells were permeabilized with BD Cytofix/Cytoperm™ solution according to the manufacturer’s instructions (BD Pharmingen), stained with antibodies, and fixed with 1% paraformaldehyde. Flow cytometry was performed using a FACSCanto (Becton Dickinson, Franklin Lakes, NJ, USA), and results were analyzed using FlowJo software (TreeStar Inc. Ashland, OR, USA).

### Enzyme-linked immunosorbent assay (ELISA) of CXCL10

Concentrations of CXCL10 in mouse sera were measured with sandwich ELISA kits (MCX100, R&D Systems, Minneapolis, MN, USA) according to the manufacturer’s instructions.

### Migration assay

Inguinal lymph node cells of CIM were harvested and the migration of lymph node cells was evaluated using a Costar Transwell system (24-well, 5-μm pore size membrane; Corning Costar, Cambridge, MA, USA). Briefly, a total of 5 × 10^5^ cells were added to the top chamber with 0.1 mL serum-free RPMI 1640. The bottom chamber was filled with 0.6 mL serum-free RPMI 1640 with or without 200 ng/mL recombinant mouse CXCL10 (R&D systems) [25]. The chambers were incubated for 3 hours at 37°C. Then, the transmigrating cells in the bottom well were counted in nine randomly captured images. Each experiment was performed in triplicate.

### Statistical analysis

All values are expressed as mean ± SD or median (minimum (min), maximum (max)). As some variables were not normally distributed, we used the *t*-test or paired *t*-test to analyze parametric variables and the Kolmogorov-Smirnov test, Mann-Whitney *U*-test, and Kruskal-Wallis test to analyze non-parametric variables (SPSS software; SPSS Inc). A *P*-value <0.05 was considered statistically significant.

## Results

### Presence of CXCL10 in the muscle and serum of CIM

**Figure 1:**
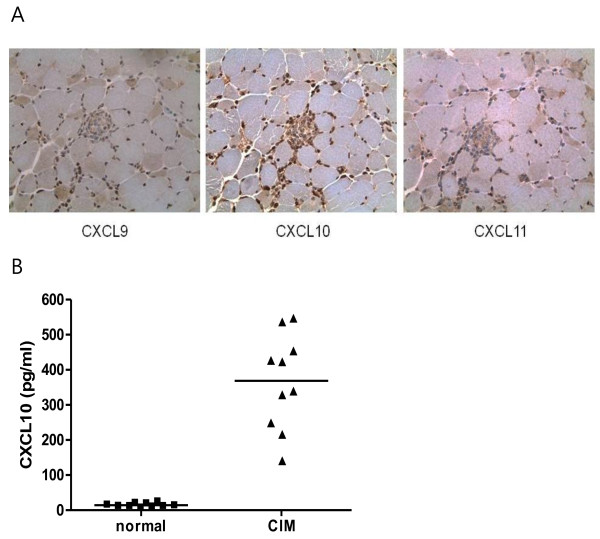
**Expression of CXCL10 in C-protein**-**induced myositis (CIM). (A)** Immunohistochemistry of CXCL9, CXCL10 and CXCL11 in the muscle of CIM (×400). In the inflammatory lesion of CIM, CXCL10 was strongly stained whereas CXCL9 or CXCL11 was weakly stained. **(B)** Serum level of CXCL10 in normal mice and CIM mice. The level of CXCL10 was measured by ELISA in the sera of normal mice (n = 10) and CIM mice (n = 10) at 3 weeks after induction. The serum level of CIM was more elevated in CIM than in normal mice (normal mouse, 14.3 ± 5.3 pg/ml versus CIM, 368.5 ± 135.6 pg/ml, *P* <0.001). The horizontal lines indicate the mean.

To investigate whether CXCL10 is expressed in CIM, we stained the muscle of CIM with anti-CXCL9, anti-CXCL10, or anti-CXCL11 antibody. Immunohistochemistry showed the positive staining of CXCL10 in the inflammatory lesion of CIM. CXCL9 or CXCL11 was weakly stained (Figure 1A). In addition, serum levels of CXCL10 were increased in CIM compared to normal mice (normal mouse, 14.3 ± 5.3 pg/mL versus CIM, 368.5 ± 135.6 pg/ml, *P* <0.001 (*t*-test) Figure 1B).

### CXCR3-positive cells in the muscle and regional lymph node of CIM

**Figure 2:**
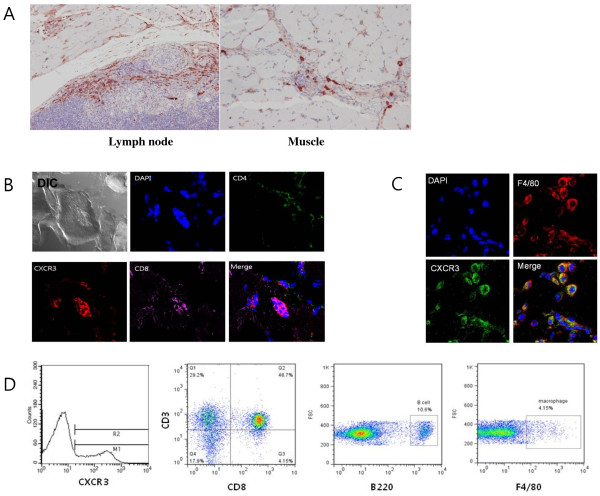
**Expression of CXCR3 in the lymph node and muscle of C-protein-induced myositis (CIM). (A)** Immunohistochemistry of CIM muscle tissue and lymph node. CXCR3 was expressed in some cells of the lymph node cells (left, ×200) and inflammatory lesion in the muscle of CIM (right, ×400). **(B)** Immunostaining of cells invading myofiber in muscle of CIM. Differential interference contrast (DIC) showed myofiber and invasive cells in the middle of myofiber. The CD8 was co-stained with CXCR3 whereas CD4 was not (×1,000). **(C)** Immunostaining of inflammation in muscle of CIM showed F4/80 co-stained with CXCR3 (×1,000). **(D)** CXCR3-positive immune cells in CIM lymph node. CIM lymph node cells were analyzed by flow cytometry. CXCR3-positive cells were gated from lymph node cells. CXCR3-positive cells were analyzed by cell-specific markers (CD3 for T cells; B220 for B cells; F4/80 for macrophages).

CXCR3 positive cells were also scattered in the lymph nodes and inflammatory lesions of muscle tissue (Figure 2A). Moreover, CXCR3-positive cells invading myofiber expressed CD8 but not CD4 (Figure 2B). F4/80+ macrophages at the focus of the inflammation, not within myofiber, also expressed CXCR3 (Figure 2C). The proportion of CXCR3 positivity in immune cells of regional lymph nodes was measured by flow cytometry. Normal mice did not show discrete lymphadenopathy, thus, lymph node cells could not be obtained. Using flow cytometry, the CXCR3+ cell was found to be 15.7 ± 3.7% among CIM lymph node cells. CXCR3+ cells were composed of CD3+CD8+ T cells (51.5 ± 3.0%), CD3+CD8- T cells (31.4 ± 2.9%), B220+ cells (12.1 ± 6.0%) and F4/80+ cells (4.3 ± 2.6%, Figure 2D). The proportion of CXCR3+ T cells among CD4+ T cells was 23.5 ± 4.7% while the proportion of CXCR3+ T cells among CD8+ T cells was 65.9 ± 2.1% (n = 6, *P* <0.001, paired *t*-test).

### IFN-γ expression increased in CXCR3 + CD8+ T cells of CIM regional lymph node

**Figure 3:**
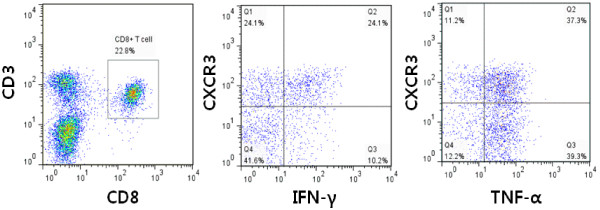
**The IFN-γ and TNF-α positivity by CXCR3 expression in CD8+ T cells.** Lymph node cells of C protein-induced myositis (CIM) were stimulated with phorbol 12-myristate 13-acetate, ionomycin and brefeldin A for 4 hours. CD3+CD8+ cells were gated (left) and analyzed according to the presence of CXCR3 and effector molecules. Representative figure of three experiments.

The intracellular cytokines IFN-γ and TNF-α in CD8+ T cells were analyzed by flow cytometry. CXCR3 positivity was associated with IFN-γ positivity (CXCR3+CD8+ T cell, 28.0 ± 4.2% versus CXCR3-CD8+ T Cell, 9.5 ± 1.5%, *P* = 0.016, paired *t*-test). TNF-α+ cells were also present. However, TNF-α was not associated with CXCR3 positivity in the lymph node cells of CIM (CXCR3+CD8+ T cell, 34.7 ± 4.3% vs. CXCR3-CD8+ T cell, 38.0 ± 1.5%, *P* = 0.362, paired *t*-test, Figure 3).

### Migration of CIM lymph node cells was increased by CXCL10

Inguinal lymph node cells of CIM were stimulated with CXCL10 (200 ng/ml) or without CXCL10 in the migration assay. The degree of migration was calculated as a chemotactic index (number of migrated cells in the presence of CXCL10/number of migrated cells in the absence of CXCL10). Increased migration of the cells in the presence of CXCL10 was observed (chemotactic index was 1.91 ± 0.45, n = 5, *P* = 0.011 versus control, Kolmogorov-Smirnov test).

### Therapeutic effect of neutralizing anti-CXCL10 antibody in CIM

**Figure 4:**
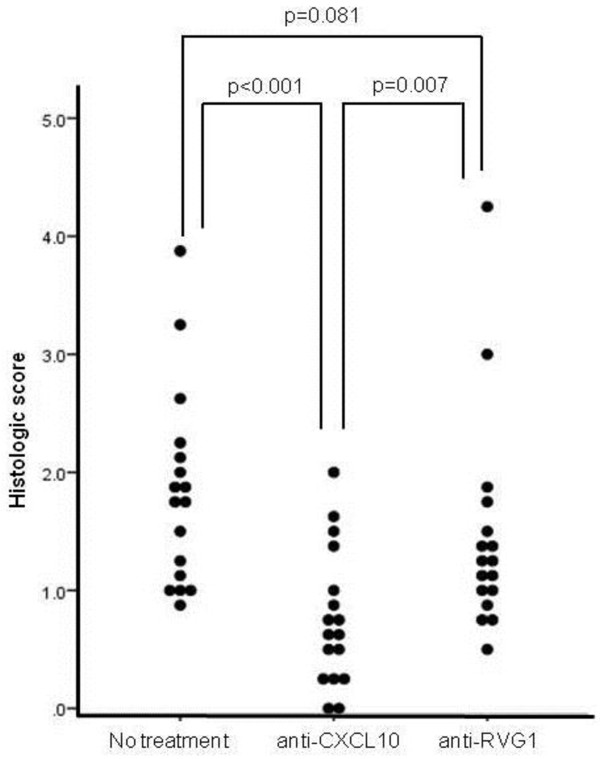
**Therapeutic effects of anti-CXCL10 or control antibody treatment in C-protein-induced myositis (CIM).** After inducing CIM, mice were treated with anti-CXCL10 antibody or control antibody (anti-RVG1) or were not treated (n = 17 per group). The group treated with anti-CXCL10 showed a lower inflammation score in muscles than those with anti-RVG1 or no treatment. No treatment: no treatment group, anti-CXCL10: anti-CXCL10 treatment group, anti-RVG1: anti-RVG1 treatment group. anti-RVG1, mouse anti-rotavirus IgG1.

The CIM mice were treated with intraperitoneal injection of monoclonal anti-CXCL10 (200 μg/100 μL) or anti-RVG1 antibody (as control, 200 μg/100 μL) every other day from day 8 to day 20. Three weeks after induction, muscle inflammation was compared between treatment groups by the histologic score. The group treated with monoclonal anti-CXCL10 antibody showed significant improvement of muscle inflammation (n = 17 per group, median (min, max), anti-CXCL10 treatment, 0.625 (0, 2.00) versus anti-RVG1 treatment, 1.25 (0.5, 4.25) versus no treatment, 1.75 (0.875, 3.875), *P* <0.001, Kruskal-Wallis test). The group treated with anti-CXCL10 was improved compared with the group treated with anti-RVG1 (*P* = 0.007, Mann-Whitney *U*-test) or the group which did not receive any treatment (*P* <0.001, Mann-Whitney *U*-test, Figure 4). In addition, serum levels of CXCL10 were not different between the group treated with anti-CXCL10 and the group treated with anti-RVG1 (n = 10, anti-CXCL10 treatment, 370.51 ± 123.39 pg/ml versus anti-RVG1 treatment, 381.12 ± 111.74, pg/mL, *P* = 0.843, *t*-test).

## Discussion

We investigated the role of the CXCL10/CXCR3 axis using a murine model of polymyositis based on a previous study on the chemokine profile of human IIM [6]. CXCL10 and CXCR3 were expressed in the inflammatory lesion in the CIM muscle tissue. Moreover, CXCR3+CD8+ T cells infiltrated myofiber. Treatment with anti-CXCL10 ameliorated muscle inflammation in CIM mice, which suggested that the CXCL10/CXCR3 interaction seems to play a crucial role in inflammatory cell migration into muscle in CIM. However, the serum level of CXCL10 was not different between anti-CXCL10 treatment group and anti-RVG1 treatment group despite efficacy of treatment. It is well known that treatment of anti-TNF agent can increase serum level of TNF-α. Serum TNF-α level in patients with various inflammatory diseases such as rheumatoid arthritis, ankylosing spondylitis or TNF receptor-associated periodic syndrome was known to be increased after treatment with soluble receptor [26] or anti-TNF antibody [27] irrespective of efficacy. The cause of elevation can be attributed to increased half-life of TNF-α [28] or upregulated expression of TNF-α [29]. Presence of anti-CXCL10 could also interfere with the CXCL10 assay [30].

Several animal models of myositis have been introduced [22,31-33]. CIM used in this study was established as a simple murine model of polymyositis. A single injection into mice of recombinant human muscle protein induced severe and clinically significant inflammation of the skeletal muscles. Previous studies on the CIM demonstrated that several types of immune cells could be involved. Macrophages and CD4+ T cells are also abundant in the muscle inflammation [22]. The depletion of CD8+ T cells or CD4+ T cells showed protective effects in CIM [22]. Thus, CD4+ T cells as well as CD8+ T cells might participate in the pathogenesis. However, CD8+ T cells were enriched in the endomysial site, the site of the muscle injury, and expressed perforins preferentially at the endomysial site. Class I major histocompatibility complex (MHC) expression was upregulated in muscles with severe inflammation in mice with CIM [22]. Moreover, removal of class I MHC significantly suppressed myositis and the adoptive transfer model suggested that the CD8 T cell-induced muscle injuries were significantly more severe than the CD4 T cell-induced muscle injuries [23]. Especially when evaluating the necrotic muscle area representing the area with direct muscle injury, CD8+ T cells were dominant. In this regard, the new CIM model provides a clear contrast to the previous experimental autoimmune myositis model in which the injury appears to be driven by CD4+ T cells. The finding that CD8+ cytotoxic T lymphocytes primarily damage the muscle fibers in CIM confirmed that CIM is the mouse myositis model most analogous to human polymyositis. The present study showed that CXCR3 was expressed in CD8+ T cells more than in CD4+ T cells in regional lymph node and moreover, muscle-invasive CD8+ T cells expressed CXCR3.

In this study, CXCR3+CD8+ T cells showed more frequent IFN-γ positivity in the inguinal lymph node. IFN-γ is important in amplification of inflammation with coordination with CXCR3 [34]. IFN-γ mediates the induction of CXCL10 and its binding to CXCR3 recruits CXCR3+ cells. These CXCR3+ T cells, in turn, produce IFN-γ. This IFN-γ-CXCR3-dependent inflammatory loop potentially may not only enhance the generation of cytotoxic T cells [35] but also enhance the increased effector response [15]. Moreover, TNF-α+ T cells were abundant in the inguinal lymph node cells in this study. TNF-α+ cells have been found to be present in the muscle tissue of IIMs [36]. TNF-α or the TNF-α-related pathway are probably important in inducing CXCL10. Recently, TNF family members, B-cell activating factor and a proliferation-inducing ligand were significantly elevated in the sera of patients with IIMs and correlated positively with CXCL10 [37]. Vitamin D receptor agonist targeting downstream of TNF-α pathway decreased the CXCL10 secretion from human fetal skeletal muscle cells [38]. Thus the TNF-α/TNF-α-related pathway can play an important role in the inflammatory mechanisms of IIMs.

As mentioned earlier, CXCR3 has at least three ligands, those are CXCL9, CXCL10, and CXCL11, and the role of its ligands in various disease models is not the same. In some inflammatory models, the requirement of one CXCR3 ligand dominates, and its deficiency cannot be compensated for by the presence of the other ligands. Although all three ligands are induced by dengue virus infection, CXCL9 and CXCL11 could not compensate for the absence of CXCL10 in *Cxcl10*-/-mice [39]. In a model that uses acute lymphocytic choriomeningitis virus infection of transgenic mice that express the glycoprotein of lymphocytic choriomeningitis virus in the cells of the islets of Langerhans, all CXCR3 ligands were upregulated in the pancreas. However, disease development was abrogated only in mice treated with CXCL10 neutralizing antibodies, whereas CXCL9 neutralizing antibodies had no effect on disease development [40]. In contrast, *Cxcr3*- and *Cxcl9*-deficient, but not *Cxcl10*-deficient MRL/lpr mice were protected from autoimmune lupus-like inflammation of the kidney [41]. Among various chemokines, CXCL10 is implicated in autoimmune pathogenesis through the initiation and maintenance of Th1 response. It appears to be related with the pathogenesis of autoimmune disease and not associated with general inflammatory conditions [17]. Not only circulating levels of CXCL10 but also the tissue expression was increased in various autoimmune diseases including rheumatoid arthritis, systemic lupus erythematosus, systemic sclerosis, type I diabetes mellitus and autoimmune thyroid disease, [4,5,42-44]. From these studies, other cytokines including CXCL9 or CXCL11 may have a role in the pathogenesis of CIM. However, we chose CXCL10 in this study because its expression is abundant in the muscle tissue of CIM in this study and polymyositis unlike CXCL9 or CXCL11 [6].

The source of CXCL10 is not clear based on this study. The previous study of the immunolocalization of CXCL10 showed expression in the inflammatory lesion and vessel, but not in muscle fiber [6]. Immunohistochemistry of CIM muscle in this study showed a similar pattern of staining in the infiltrating cell. However, it might be expressed in inflamed muscle tissue. Recently, primary human muscle cell was found to secrete CXCL10 after stimulation with TNF-α or IFN-γ [45]. Those results suggested the active role of muscle cells in the immune response. Further study focusing on the interaction between muscle cell and immune cells such as CD8+ T cells, CD4+ T cells, or macrophage may be useful.

IIM is known to be a Th1-driven autoimmune process characterized by significant inflammatory cell infiltrates in muscle and other tissue, resulting in muscle injury [46]. About 25% of IIM patients cannot tolerate or are refractory to conventional therapies [47] and there are no defined guidelines for treatment of refractory myositis [48]. Therefore, the development of new therapeutic agents is necessary. In addition to polymyositis, the CXCL10/CXCR3 axis was also reported to be involved in inclusion body myositis and dermatomyositis. CXCL10 is abundantly expressed in macrophages and T cells surrounding and invading non necrotic muscle fibers in inclusion body myositis [49]. CXCL10 expression on T cells in the perimysial infiltrates of dermatomyositis and CXCR3 expression on the majority of T cells in dermatomyositis were also reported [21]. Juvenile-type dermatomyositis also showed high expression of CXCL10 in muscle tissue [50] and the expression of CXCL10 and recruitment of CXCR3+ T cells were detected in the skin lesions of dermatomyositis [51].

In clinical aspects, the development of a therapeutic agent against CXCL10/CXCR3 in IIM is plausible. As mentioned in the introduction, the studies demonstrated that the expression of CXCL10 and CXCR3 was increased in the collagen-induced arthritis model, and neutralizing anti-CXCL10 antibodies ameliorated disease manifestation in these models [52,53]. Moreover, CXCL10 and CXCR3 expression is also increased in the synovial membrane of rheumatoid arthritis patients [3,10]. Thus, a clinical trial of blocking antibody against CXCL10 showed a promising outcome [30]. Taking the results of this study into consideration in parallel with the studies on rheumatoid arthritis, the CXCL10/CXCR3 pathway may be a candidate as a therapeutic target in human IIMs.

## Conclusion

Our study reveals that expression of CXCL10 and its receptor, CXCR3 is increased in the inflammation site and lymph node cells of the CIM model. Moreover, myofiber-invasive CD8+ T cells express CXCR3. CXCL10 blockade with monoclonal antibody suppresses inflammation in muscle, which suggests CXCL10 inhibition could be used as a potential therapeutic strategy for treatment of myositis.

## Abbreviations

anti-RVG1: mouse anti-rotavirus IgG1; APC: allophycocyanin; CFA: complete Freund’s adjuvant; CIM: C-protein-induced myositis; CXCL10: C-X-C motif chemokine 10; CXCL11: C-X-C motif chemokine 11; CXCL9: C-X-C motif chemokine 9; CXCR3: C-X-C motif chemokine receptor 3; DAB: 3,3-diaminobenzidine tetrahydrochloride; ELISA: enzyme-linked immunosorbent assay; FITC: fluorescein isothiocyanate; IFN-γ: interferon-γ; IIM: idiopathic inflammatory myopathy; IP-10: Interferon-inducible protein-10; MHC: major histocompatibility complex; PBS: phosphate-buffered saline; PE: phycoerythrin; PerCP: peridinin chlorophyll; PMA: phorbol 12-myristate 13-acetate; PT: pertussis toxin; RPMI: Roswell Park Memorial Institute medium; Th1: T helper 1; TNF-α: tumor necrosis factor-α.

## Competing interests

The authors declare that they do not have any competing interests.

## Authors’ contributions

JK and JYC performed experimentation, contributed to the design of the study and wrote the draft of the manuscript. SHP, SHY, and JAP were involved in the acquisition, analysis and interpretation of data. KS, EYL, and HKa are responsible for the study design and data analysis. HKo is responsible for study design and data analysis and revising the manuscript. YWS responsible for study design, data acquisition and analysis and drafting and revising the manuscript. All authors have read and approved the manuscript for publication.
